# Efficient *Agrobacterium*-Mediated Transformation of the Commercial Hybrid Poplar *Populus Alba × Populus glandulosa* Uyeki

**DOI:** 10.3390/ijms20102594

**Published:** 2019-05-27

**Authors:** Chengwei Song, Liang Lu, Yayu Guo, Huimin Xu, Ruili Li

**Affiliations:** 1 Beijing Advanced Innovation Center for Tree Breeding by Molecular Design, Beijing Forestry University, Beijing 100083, China; songchengwei@bjfu.edu.cn (C.S.); luliang@bjfu.edu.cn (L.L.); guoyau1510@bjfu.edu.cn (Y.G.); 2 College of Biological Sciences and Technology, Beijing Forestry University, Beijing 100083, China; 3 College of Life Sciences, Peking University, Beijing 100871, China; xuhm@pku.edu.cn

**Keywords:** *Agrobacterium tumefaciens*, leaf explants, regeneration transformation, *P. alba × P. glandulosa* Uyeki

## Abstract

Transgenic technology is a powerful tool for gene functional characterization, and poplar is a model system for genetic transformation of perennial woody plants. However, the poplar genetic transformation system is limited to a number of model genotypes. Herein, we developed a transformation system based on efficient *Agrobacterium*-mediated transformation for the hybrid poplar *Populus Alba × Populus glandulosa* Uyeki, which is a fast-growing poplar species that is suitably grown in the northern part of China. Importantly, we optimized many independent factors and showed that the transformation efficiency was improved significantly using juvenile leaf explants. Explants were infected by an *Agrobacterium* suspension with the OD_600_ = 0.6 for 15 min and then co-cultured in dark conditions for 3 days. Using the improved transformation system, we obtained the transgenic poplar with overexpression of *β-glucuronidase* (*GUS*) via direct organogenesis without callus induction. Furthermore, we analyzed the *GUS* gene in the transgenic poplars using PCR, qRT-PCR, and GUS staining. These analyses revealed that the *GUS* gene was efficiently transformed, and it exhibited various expression levels. Taken together, these results represent a simple, fast, and efficient transformation system of hybrid poplar plants. Our findings may facilitate future studies of gene functions in perennial woody plants and tree breeding via transgenic technology assisted design.

## 1. Introduction

Poplar is an important group of cultivated tree species used in both ecological and economic plantations. Its popularity is largely due to its adaptability to diverse environmental conditions and its capacity for rapid growth [1]. Tree breeding by transgenic technology assisted design is the major technique for improving varieties of poplar with superior hereditary characteristics. This technique can overcome the difficulties associated with the breeding of woody plants, which require many years to produce progeny [2]. The genetic transformation system can introduce foreign genes into plants for understanding and controlling plant gene expression, which provide a powerful tool for analyzing the function of genes and the functional characterization of gene products. Then the in-depth knowledge of gene function is effective for making more purposeful and systematic breeding programs directed at transgenic technology assisted breeding of poplar.

Strategies for variety-independent genetic transformation of plants have been developed, including *Agrobacterium*-mediated transformation, viral transformation, electroporation of protoplasts, and particle bombardment [3,4,5,6]. Although both biological and physical methods have been used, the *Agrobacterium*-mediated transformation is the predominant method for poplar [7]. Since the first successful poplar transformation [3], a series of *Agrobacterium*-mediated poplar transformations have been reported, such as *P. tremula*, *P. tremuloides*, *P. alba*, *P. nigra*, *P. tremula × P. tremuloides*, *P. trichocarpa*, *P. trichocarpa × P. deltoids*, *P. deltoides × P. euramericana* [8,9,10,11,12,13,14,15].

The in vitro tissue-to-plant regeneration system is an integral part of genetic transformation procedures. Two tissue culture regeneration systems have been used in poplar: organogenesis and somatic embryogenesis. Regeneration through organogenesis is an efficient method for in vitro production of whole plants [16]. For in vitro regeneration of poplar through organogenesis, different explants have been generally used for transformation, including leaves [15,17,18], petiole [19], stems [20,21,22], roots [20], and shoot tips [23]. Interestingly, the leaf disc transformation method is the predominant method used in poplar transformation systems. In the past, new transgenic poplars with resistance to pests [24], resistance to stress [25], and modifications to wood properties [26] have been bred from different poplar species with various transformation systems. Efficient and reproducible *Agrobacterium*-mediated transformation and regeneration systems have been obtained in many poplar species. However, these have been reported only for a limited number of model genotypes, and the process is still difficult in many poplar species [16,27].

In the present study, we developed a simple and efficient system for transformation, selection, and regeneration based on *Agrobacterium*-mediated transformation of hybrid poplar *P. alba × P. glandulosa* Uyeki, known as suwon poplar. This is one of the major poplar species in Korea, and suitable for reforestation of hillsides. *P. alba × P. glandulosa* was introduced to China in 1984, and commonly used in China due to its high wood quality, fast growth, and relatively high drought tolerance. *P. alba* × *P. glandulosa* is ideal as a model plant and as a source of laboratory research materials due to its easy cultivation, rapid growth, and strong in vitro regeneration frequency characteristics. Herein, we optimized many independent factors to achieve a stable regeneration efficiency of poplar. We used *β-glucuronidase* (*GUS*) as a reporter gene, which has proved to be efficient in vivo and achieved the transgenic poplar with this method. Furthermore, we used PCR, quantitative real-time PCR (qRT-PCR), and β-glucuronidase (GUS) staining to examine the expression levels of the target *GUS* gene in the wild-type and transgenic poplar. The purpose of this study is to establish a simple, fast, and stable transformation system for *P. alba × P. glandulosa*. The results of this study should provide a powerful tool for the study of gene functions in perennial woody plants.

## 2. Results

### 2.1. Optimization of the Transformation Procedure

**Figure 1:**
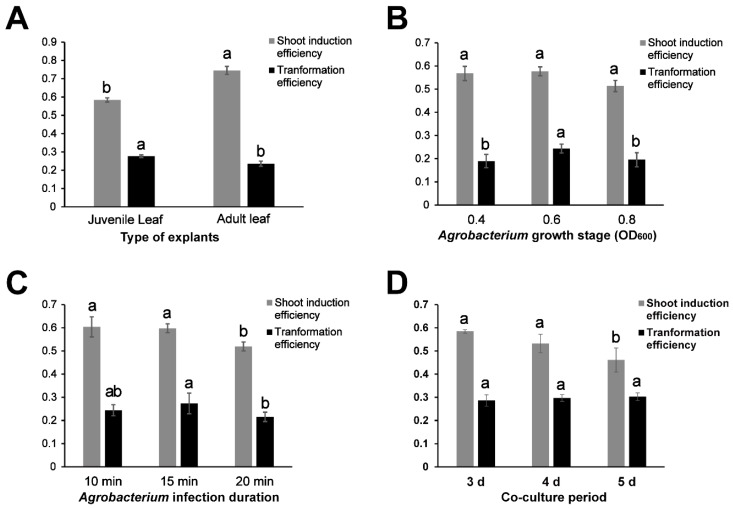
Factors that affect the shoot induction efficiency and transformation efficiency of *P. alba × P. glandulosa*. (**A**) Ages of leaves. (**B**) Growth stages of *Agrobacterium* bacteria. (**C**) *Agrobacterium* infection duration. (**D**) Co-culture period. a and b showed the statistical significance of differences that determined using the Student’s *t*-test (*p* < 0.05).

To establish the transformation system of *P. alba × P. glandulosa*, we optimize several operations in the transformation procedure, including the age of leaf explants (Figure 1A), the growth stage of bacterium (Figure 1B), *Agrobacterium* infection duration (Figure 1C), and co-culture period (Figure 1D). The transformation efficiency was significantly increased with juvenile leaf explants, but the frequency of shoot induction was simultaneously decreased (Figure 1A). The transformation efficiency was significantly increased when *Agrobacterium* infection with an OD_600_ of 0.6, and the transformation efficiency was decreased when the growth time of *Agrobacterium* was excessively long or short (Figure 1B). The highest transformation efficiency was obtained with an *Agrobacterium* incubation time of 15 min (Figure 1C). The transformation efficiency was not significantly different with different co-culture periods, but the shoot induction efficiency significantly decreased with the extension of the co-culture period (Figure 1D).

### 2.2. Simple and Fast Transformation System

**Figure 2:**
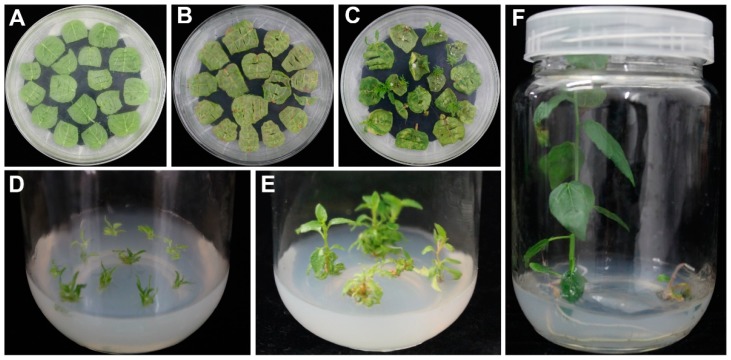
The flowchart of the transformation system for *P. alba × P. glandulosa.* (**A**) Pre-culture of leaf explants on M1. (**B**) Shoot induction of leaf explants on M3 after co-cultivated with *Agrobacterium* bacteria. (**C**) Shoot induction of leaf explants on M3 for 4 weeks. (**D**) Shoots cultured on M4 for root induction. (**E**) Root induction on M4 for 2 weeks. (**F**) Transgenic poplar seedlings culture on M4.

A simple and fast *Agrobacterium*-mediated transformation system was developed for *P. alba × P. glandulosa* using leaf explants and the following protocol: 2 days of pre-culture (Figure 2A), 3 days of co-culture, 4 to 5 weeks of shoot induction (Figure 2B,C), and 4 to 5 weeks for root induction (Figure 2D,E) and seedlings development (Figure 2F). In addition, to determinate the stability of kanamycin-resistance in regenerated poplar plants, the regenerated poplar plants were subcultured on M4 for kanamycin-resistance plants propagation and showed that 96% of the explants from regenerated poplar plants developed into whole poplar plants. Furthermore, to ensure that the regenerated poplar plants were free from *Agrobacterium*, we subcultured the regenerated poplar plants on M1 for plant propagation free from kanamycin and showed no *Agrobacterium* occurred on the culture medium or plants in the whole process of subculture. During transformation experiments, a total of 115 leaf segments of *P. alba × P. glandulosa* were infected with *Agrobacterium*, 63 shoots were induced cultured on shoot-inducting medium M3, 39 independent kanamycin-resistant plants were obtained cultured on root-inducting medium M4, and 37 positive transgenic poplar lines were finally obtained after being subcultured on M4. The results indicate that the transformation frequency of the system was 32.18%.

### 2.3. Positive Plant Identification by PCR Analysis

**Figure 3:**
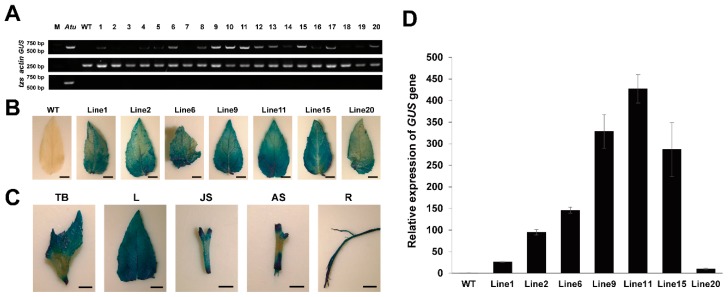
PCR, qRT-PCR, and GUS staining analysis of transgenic poplars. (**A**) PCR product of 35S-GUS with 680 bp was detected in *Atu,* and all of the transgenic poplars, PCR product of *actin* gene with 250 bp was detected in all of the poplars, and PCR product of *tzs* gene with 660 bp was detected in *Atu*. M, Marker; *Atu*, *Agrobacterium tumefaciens*; WT, wild type of *P. alba × P. glandulosa*; 1–20, transgenic poplars. (**B**) GUS staining of different transgenic poplar and WT plants. WT, wild type; Line 1–Line 7, different lines of transgenic poplars. Bars = 0.5 cm. (**C**) GUS staining of different explants of Line 6. TB terminal bud; L, leaf; JS, juvenile stem; AS, adult stem; R, root. Bars = 0.5 cm. (**D**) qRT-PCR analysis of transgenic poplar and WT plants. WT, wild type; Line 1–Line 7, different lines of transgenic poplars.

To determine whether the transgenic poplar plants were *GUS*-positive, we selected 20 transgenic poplar plants and isolated the genomic DNA from leaves for PCR analysis. The 35S promoter and part of the *GUS* gene were PCR-amplified using a specific primer pair and the expected band size of 680 bp, the *actin* gene that existing in poplar was used as an internal control. The bands were observed at the expected size from all of the samples, but they were not observed for the wild-type poplar plant (Figure 3A). These results showed that all 20 poplar plants are transgenic plants. To ensure that the regenerated poplar plants were free from *Agrobacterium*, we used the *tzs* gene that does not belong to the T-DNA region in *Agrobacterium* strain as a positive control. The bands of *tzs* gene were observed from *Agrobacterium* strain, but they were not observed in all of the poplar plant samples, including the wild-type poplar plant and all of the transgenic poplar plant. (Figure 3A).

### 2.4. Quantitative Assay of GUS Expression by qRT-PCR

To analyze the *GUS* expression level of different lines, we isolated the genomic RNA from the randomly selected leaves of the seven positive transgenic poplar plants and wild-type plants. We then used qRT-PCR to analyze the relative expression of *GUS*. The expression of *GUS* varied from 10 times higher to 427 times higher than the level of wild-type plants. This result confirmed that the expression of *GUS* differed among the various transgenic lines (Figure 3D).

### 2.5. β-Glucuronidase Histochemical Assays

We further stained the leaves of the seven poplar plants and wild-type plants that were analyzed via qRT-PCR. The GUS staining of leaf explants of transgenic poplars showed various levels of intensity, which suggests that the expression of GUS was different in various transgenic poplar plants (Figure 3B). In addition, we stained different explants of the transgenic poplar line 6, including terminal bud, leaf, juvenile stem, adult stem, and root. All of the explants showed positive GUS staining (Figure 3C).

## 3. Discussion

Genetic transformation is a powerful tool in plant molecular biology and functional genomics research. Since the first successful poplar transformation was established [3], poplars have been transformed for genetic improvement and gene function research far more than any other forest tree species [7,27]. A large number of factors affect the transformation efficiency and the regeneration during the process of poplar genetic transformation, including the explant source, the growth stage of bacterium, co-culture time period, and *Agrobacterium* infection duration. Juvenile sources of explant tissues have superior regeneration potential compared with other sources due to their lower lignin content [17]. Moreover, *Agrobacterium* concentration and infection time significantly affect the transformation efficiency [15,17]. In this study, we analyzed the poplar transformation efficiency by varying the developmental stage of leaf explants, *Agrobacterium* concentrations, the duration for the co-culture period, and duration of *Agrobacterium* infection. These results indicated that the development stage of leaf explants was the main influencing factor for transformation efficiency of *P. alba × P. glandulosa*. In addition, the best results for *P. alba × P. glandulosa* genetic transformation were obtained using juvenile leaf explants that were dipped into *Agrobacterium* suspension (OD_600_ = 0.6) for 15 min and then co-cultured in dark conditions for 3 days (Figure 1).

A major problem for the poplar transformation system is inducing shoots from transformed cells, and there are two major approaches for shoot induction in most of the existing poplar transformation systems. One method is inducing cell division and callus growth on the cut surface of poplar explants infected with *Agrobacterium* and then inducing shoots from induced calluses [22]. Callus induction is a time-consuming and complex operation, and methods that include the callus phase may lead to plants that differ from the mother plant due to somaclonal variations [28]. Another method is directly inducing shoots from the cut poplar explants infected with *Agrobacterium* [18,29]. In the present study, we developed a fast poplar transformation system for *P. alba* × *P. glandulosa* via direct organogenesis without callus induction (Figure 2). The whole genetic transformation process was completed within 3 months. In addition, the regenerated poplar plants were subcultured on M4 and M1, respectively, showing that the kanamycin-resistance transgenic poplar plants were subcultured steady and the transgenic poplar plants were free from *Agrobacterium* contamination. These results suggested that the *Agrobacterium*-mediated transformation regeneration system was adopted as the fast and effective for *Populus. alba* × *P. glandulosa*. Nonetheless, the transformation system of *P. alba* × *P. glandulosa* exhibits a relatively higher transformation frequency at 32.18%.

In plant genetic transformation, the transgenic tissues will form chimeric shoots that contain transgenic and non-transgenic sections with a certain probability [30,31]. PCR is a powerful and direct tool for detecting a target gene that has been inserted into the genome, and it is routinely used in the identification of transgenic plants [18]. In the present study, transgenic poplar plants that subcultured two times were detected by the previously optimized vector-specific genomic PCR, This indicates that the *GUS* gene was inserted into the poplar genome successfully in the 20 transgenic poplar lines. (Figure 3A).

Transgene expression is usually correlated to the transgene copy number that was inserted into the host plants genome [32,33]. The qRT-PCR method has been validated for accurate estimation of transgene expression in various transgenic plants [34]. The current results showed various expressions of *GUS* in the seven samples. These levels are significantly higher than levels for wild-type plants, revealing that this transformation system can transform the exogenous gene into *P. alba × P. glandulosa* efficiently, and increased the gene expression significantly (Figure 3D). In addition, the *GUS* histochemical analysis showed that the high expression of the *GUS* gene led to high staining intensity for different transgenic poplar plants and different explants, indicating that the staining intensity was consistent with the *GUS* gene expression (Figure 3B,C). Together, these results provide strong evidence that transformation systems are efficient for *P. alba × P. glandulosa*. Additionally, the transformed exogenous gene is suitable to achieve high expression levels of the target gene and protein.

## 4. Materials and Methods

### 4.1. Plant Materials and Growth Conditions

Explants were collected from *P. alba × P. glandulosa* plants, sterilized with 0.2% NaClO for 15 min, rinsed three times with sterile water, and transferred to the culture bottle. Seedlings of poplar (*P. alba × P. glandulosa*) were cultured on M1 (Table 1) and subjected to a 16-h light:8-h dark photoperiod with 500 to 600 μmol m^−2^ s^−1^ of photosynthetically active radiation. Plants were kept at 25 °C during the day and at 22 °C at night.

### 4.2. Agrobacterium Culture and Transformation Procedure

We constructed the *35S:GUS* vector and transformed to *Agrobacterium tumefaciens* GV3101 [35]. A single colony of *Agrobacterium tumefaciens* was grown in LB medium (containing 50 mg/L kanamycin and 50 mg/L rifampicin) overnight at 28 °C with shaking until the culture density reached an OD_600_ of 0.6. The *Agrobacterium tumefaciens* cells were harvested by centrifugation at 5000 rpm for 10 min. The cells were then suspended with 1/2 MS solution (pH 5.8 to 6.0) containing 5% (*w*/*v*) sucrose and acetosyringone (100 μM) as the transformation solution.

For the whole process of genetic transformation, the explants were cultured on Murashige and Skoog medium with different supplementary components, as shown in Table 1. The leaf explants excised from 3-week-old plantlets were cut with three wounds perpendicular to main veins and cultured on M1 for 2 days. The cut leaves were incubated with the suspended *Agrobacterium tumefaciens* for 15 min with slow shaking every 5 min. The cut leaves were blotted with sterile filter paper to remove the excess bacteria and then cultured on M2 in dark conditions for 3 days. The co-cultivated leaves were washed 2 times in sterilized distilled water for 5 min, blotted with sterile filter paper to remove the excess water, and transferred to M3 for shoot induction. The co-cultivated leaves were transferred to fresh M3 every 10 days, and the growth conditions were similar to the growth conditions of the *P. alba × P. glandulosa* plants. The shoots grew to 1 to 2 cm after 4 weeks, and they were then cut off and transferred to M4. In this medium, the roots formed and the whole poplar plants were developed. The transgenic poplar plants were grown on M1 for 3 weeks to ensure that plants were free from *Agrobacterium* contamination.

### 4.3. Plant Genomic DNA Isolation and PCR Analysis

The genomic DNA was isolated from leaves of transgenic and wild-type poplar plants via the cetyltrimethylammonium bromide (CTAB) procedure described previously, with some modifications [36]. For PCR analyses, a forward primer (5′-CCTCTGCCGACAGTGGTC-3′) was designed from the 35S promoter, and a reverse primer (5′-CATCGGCTTCAAATGGCGTATAGC-3′) was designed from *GUS*. For an internal control, *actin* gene that existing in poplar was used [37], and its primers were forward 5′-AAGGTTGTTGCACCACCAGA-3′ and reverse 5′-AACACACAGTCCATCACCGC-3′. The primers of *tzs* gene were forward 5′-TCTGGCCACTGAGGAAAATC-3′ and reverse 5′-ATCTACGGACCGACTTGCAG-3′ [38].

### 4.4. Quantitative Analysis of GUS Expression by qRT-PCR

Whole seedlings of poplar were collected and stored in liquid nitrogen. The total RNA of these plants was isolated using an RNA Isolation Kit (TIANGEN, Beijing, China). Reverse transcription was performed using the First-Strand cDNA Synthesis Kit (TIANGEN, Beijing, China). qRT-PCR was performed using the SYBR Green Mix (TAKARA) in an optical 96-well plate. The primers for *GUS* were forward 5′-CTGTGGAATTGATCAGCGTTGGTGG-3′ and reverse 5′-AAGACTTCGCGCTGATACCAGACG-3′. For an internal control, *18SrRNA* was used, and its primers were forward 5′-CGAAGACGATCAGATACCGTCCTA-3′ and reverse 5′-TTTCTCATAAGGTGCTGGCGGAGT-3′. The initial denaturing time was 94 °C for 30 s, followed by 40 cycles at 95 °C for 15 s, 60 °C for 15 s, and 72 °C for 15 s, with a final extension at 72 °C for 10 min.

### 4.5. β-Glucuronidase Histochemical Assay

The samples were thoroughly rinsed with distilled water and vacuumed for 20 min while immersed in GUS staining solution and then incubated overnight at 37 °C. The GUS staining solution consisted of 10 mM Na_2_EDTA, 100 mM phosphate buffer (pH 7.0), 2 mM K_4_Fe(CN)_6_, 2 mM K_3_Fe(CN)_6_, and 1 mg/mL of X-Gluc (5-bromo-4-chloro-3-indolyl β-d-glucuronide). The stained samples were soaked in 70% (*v*/*v*) ethanol to remove chlorophyll.

## 5. Conclusions

In summary, we optimized many factors in the genetic transformation process of *P. alba × P. glandulosa*, and the transformation efficiency was improved significantly. We developed a simple and fast poplar transformation system for *P. alba × P. glandulosa* with directly induced shoots. Furthermore, the PCR, qRT-PCR, and GUS staining results showed that the transformation systems are efficient for *P. alba × P. glandulosa* with a 32.2% transformation frequency.

## Figures and Tables

**Table 1:** Composition of media for cultivation, transformation, selection, and regeneration of hybrid poplar *P. alba × P. glandulosa*.

Components	M1 (Plant Propagation and Pre-Culture)	M2 (Co-Culture)	M3 (Shoot Induction)	M4 (Root Induction)
MS	4.43 g/L	4.43 g/L	4.43 g/L	2.215 g/L
Sucrose	30 g/L	30 g/L	30 g/L	30 g/L
Agar	5.8 g/L	5.8 g/L	5.8 g/L	5.8 g/L
NAA	—	0.05 mg/L	0.05 mg/L	0.02mg/L
6-BA	—	0.5 mg/L	0.5 mg/L	—
IBA	—	—	—	0.05 mg/L
Kanamycin	—	—	30 mg/L	30 mg/L
Cefotaxime	—	—	200 mg/L	200 mg/L
Timentin	—	—	200 mg/L	200 mg/L
pH	5.8	5.8	5.8	5.8
